# Are Rural and Urban Emergency Departments Equally Prepared to Reduce Avoidable Hospitalizations?

**DOI:** 10.5811/westjem.2019.2.42057

**Published:** 2019-04-16

**Authors:** Margaret B. Greenwood-Ericksen, Michelle L. Macy, Jason Ham, Michele M. Nypaver, Melissa Zochowski, Keith E. Kocher

**Affiliations:** * University of New Mexico, Department of Emergency Medicine, Albuquerque, New Mexico; † Ann & Robert H. Lurie Children’s Hospital of Chicago and Feinberg School of Medicine, Northwestern University, Chicago, Illinois; ‡ University of Michigan, Department of Internal Medicine, Ann Arbor, Michigan; § University of Michigan, Department of Emergency Medicine, Ann Arbor, Michigan; ¶ University of Michigan, Institute for Healthcare Policy and Innovation, Ann Arbor, Michigan; || University of Michigan, Department of Pediatrics, University of Michigan, Ann Arbor, Michigan; # University of Michigan, College of Engineering, XTRM Labs, Ann Arbor, Michigan

## Abstract

**Introduction:**

Attempts to reduce low-value hospital care often focus on emergency department (ED) hospitalizations. We compared rural and urban EDs in Michigan on resources designed to reduce avoidable admissions.

**Methods:**

A cross-sectional, web-based survey was emailed to medical directors and/or nurse managers of the 135 hospital-based EDs in Michigan. Questions included presence of clinical pathways, services to reduce admissions, and barriers to connecting patients to outpatient services. We performed chi-squared comparisons, regression modeling, and predictive margins.

**Results:**

Of 135 EDs, 64 (47%) responded with 33 in urban and 31 in rural counties. Clinical pathways were equally present in urban and rural EDs (67% vs 74%, p=0.5). Compared with urban EDs, rural EDs reported greater access to extended care facilities (21% vs 52%, p=0.02) but less access to observation units (52% vs 35%, p=0.04). Common barriers to connecting ED patients to outpatient services exist in both settings, including lack of social support (88% and 76%, p=0.20), and patient/family preference (68% and 68%, p=1.0). However, rural EDs were more likely to report time required for care coordination (88% vs 66%, p=0.05) and less likely to report limitations to home care (21% vs 48%, p=0.05) as barriers. In regression modeling, ED volume was predictive of the presence of clinical pathways rather than rurality.

**Conclusion:**

While rural-urban differences in resources and barriers exist, ED size rather than rurality may be a more important indicator of ability to reduce avoidable hospitalizations.

## INTRODUCTION

Emergency departments (ED) play a critical role in the delivery of acute ambulatory and inpatient care. EDs now serve as the primary source of hospital admissions^1^,^2^ and increasingly serve as a hub for unplanned acute care needs.^3^,^4^ As emergency providers are on the frontlines of admission decisions, their ability to identify opportunities for outpatient pathways as alternatives to an admission is critical to optimizing hospitalization practices. In other clinical contexts, low-value care has been defined as patient care that provides no net health benefit.^5^ Similarly and in the context of this work, low-value hospitalizations are conceptualized as those admissions that are unlikely to provide an overall benefit, particularly when safe and effective outpatient alternatives exist. Avoiding such hospitalizations can reduce costs and potentially improve longer term population health outcomes by preventing the exposure to adverse events tied to the inpatient setting.

In efforts to improve the integration of care delivery within a local health system and better use of alternative pathways to hospitalization, some EDs and their hospitals have invested resources in comprehensive care coordination efforts.^6^–^8^ EDs may embed personnel such as care managers and discharge planners to support this work. EDs have also developed clinical pathways to standardize care, frequently specifying criteria to determine safe disposition to hospital inpatient or observation unit vs home with additional services. These clinical pathways commonly include mechanisms to accelerate outpatient follow-up in an effort to reduce reliance on inpatient admissions for consultations and tests that can be obtained in an outpatient setting.^9^ However, to date the majority of publications describing such innovations are from urban, suburban, and academic EDs,^10^–^13^ and therefore little is known about the presence of pathways to avoid low-value admissions in community and rural EDs.

As rural populations are health disparity populations, studying rural populations and their sites of emergency care delivery is critical to understanding and improving rural health outcomes. Rural populations are of particular interest as they may be at higher risk for low-value admissions from the ED as a result of several factors. Rural patient populations tend to be older, with more chronic conditions^14^ and less access to primary care^15^,^16^ when compared to urban populations. As a result, rural areas may have fewer resources by which to reduce avoidable admissions. To explore this hypothesis, we conducted a cross-sectional, web-based survey of hospital-based EDs in Michigan. We examined differences in the availability of pathways to avoid low-value admission from the ED, as well as resources available in the community that may prevent these admissions.

## METHODS

### Subjects

We developed a list of all 135 hospital-based EDs in the state of Michigan. Contact information for medical directors and/or nurse managers was collected through professional relationships and web-based searches conducted by the study team.

**Population Health Research Capsule**

What do we already know about this issue?

*Emergency departments (EDs) serve as the primary source of hospitalizations. Optimizing these practices requires identifying opportunities for alternative outpatient pathways of care*.

What was the research question?

What are the perceived barriers and availability of alternative pathways to hospitalization from the ED and are there urban vs rural differences?

What was the major finding of the study?

*Both urban and rural EDs have implemented alternative pathways but confront challenges related to social support, and patient and provider preferences*.

How does this improve population health?

*Perceived poor integration of the ED into outpatient settings limits the success of building alternatives to hospitalization programs for both urban and rural communities*.

### Survey Development

This study was performed by the coordinating center of the Michigan Emergency Department Improvement Collaborative (MEDIC). MEDIC is a physician-led, collaborative, quality improvement network supported through a partnership with Blue Cross Blue Shield of Michigan and Blue Care Network within the Value Partnerships program.^17^,^18^ MEDIC measures performance relative to evidence-based, consensus quality goals across several domains to improve outcomes. One of the unique quality initiatives within MEDIC is the Program on Alternatives to Hospitalization (M-PATH). This program works with MEDIC partner hospitals and providers throughout the state of Michigan to support the development, implementation, and evaluation of clinical pathways designed to improve the quality and value of admission decisions made in the ED.

The M-PATH team designed an online survey as part of an environmental scan to inform future quality improvement efforts by understanding the scope of the problem of avoidable admissions and use of clinical pathways to guide admission decisions. The target study population was medical directors and/or nurse managers at all 135 EDs in the state of Michigan. The institutional review board of the University of Michigan approved this study.

The survey contained 14 questions developed by a team of emergency physicians and health services researchers (Appendix). Questions were structured with fixed-choice responses and a free-text option for “other” responses. The survey was designed to be completed in less than 15 minutes. Questions explored the use of clinical pathways and protocols for ED care, factors contributing to the decision to admit a patient from the ED, hospital and community resources available to avoid hospitalization, and hospital characteristics including annual ED visits and number of ED beds. We inquired specifically about the presence of diagnosis- or complaint-related clinical pathways or protocols. (Examples provided included asthma, atrial fibrillation, chest pain, and head injury.)

Questions also requested information on the presence of community or health system standardized services (including extended care facilities, wound care, observation units, home healthcare and rapid follow-up to primary or specialist care), along with resources available to reduce/avoid admission, barriers in connecting patients to outpatient services (such as lack of family and/or social support, primary care/specialty care preference for admission, lack of time/support for care coordination, and lack of timely access to outpatient or home-care services), and individuals who may influence admission decisions (primary care, specialists, physical therapists, ED-based pharmacists, and care coordinators). We also asked respondents for information on the number of annual ED visits, number of ED beds, staffing model, and typical ED boarding times at their facility.

### Survey Testing

After initial survey development, we conducted pilot testing of the survey to ensure clarity of the questions and response options with three individuals from within the state of Michigan and six individuals outside of Michigan representing backgrounds in general and pediatric emergency medicine and general emergency medicine as well as expertise in research or leadership in observational medicine. The survey was refined based on the feedback from pilot testing.

### Survey Administration

Surveys were distributed via email with an embedded link to the medical directors and/or nurse managers of each ED in Michigan. The first request for participation was sent in late July 2016 and up to three reminder messages were sent over the subsequent eight weeks to those subjects who did not complete the survey. We used the Qualtrics (Provo, Utah) platform for survey administration and data collection.

### Data Analysis

We performed descriptive statistics and tests of significance where appropriate using chi-squared analysis. In our analysis of barriers to connecting patients to outpatient services, we defined the presence of five or more of the eight answer choices as clinically significant, as this would represent a majority of barriers being selected. We categorized EDs as urban or rural by their county location in accordance with the Office of Management and Budget definitions (OMB). Those EDs in metropolitan statistical areas were categorized as urban, with micropolitan and non-metro categorized together as rural.

In our multivariable analysis, we constructed logistic regression models to determine if rurality predicted the primary outcome of presence of clinical pathways. Models adjusted for the following covariates: presence or absence of key healthcare access indicators of timely outpatient primary care and specialty care follow-up; outpatient resources such as wound care or home healthcare; and presence of significant barriers to avoiding admission, which was defined as hospitals reporting the presence of five or more of the eight answer choices (the majority). Additionally, the models were adjusted for the average ED boarding time (continuous variable), and number of ED beds (continuous variable). Finally, we also included the staffing model (hospital employee or contracted physician group) as a covariate given its hypothesized influence on hospitalization decisions, as these arrangements could correlate with particular financial incentives and familiarity with local protocols. We assessed whether to also include the covariate of annual ED visit volume but found it to be collinear with number of ED beds. Statistical significance was set at 0.05; we analyzed all data from the surveys using STATA (Version 14, College Station, Texas).

## RESULTS

### Description of Emergency Departments

Of the 135 hospital-based EDs, we received responses for 64 (47%). Of these, 33 were classified as urban and 31 were classified as rural in accordance with the OMB definition. ED characteristics are displayed in Table 1.

### Presence of Clinical Pathways and Programs to Reduce Admission

The presence of pathways to guide admission decisions was reported at 45% of all respondent EDs, without significant difference between rural and urban centers (41.4% vs 58.6%; p=0.304). Most EDs (74.2% rural and 66.7% urban) reported the presence of one or more standardized programs or services designed to reduce avoidable inpatient admissions (Table 2). Of these standardized programs and services, wound care (62.5% vs 33.3%, p=0.028) and extended care facilities (52.2% vs 21.4%, p=0.022) were more likely to be reported in rural compared with urban EDs. In contrast, observation units were less likely in rural compared with urban EDs (35.4% vs 51.5%, p=0.042). Same or next day access to primary care follow-up was uncommon overall (23.8%) with 34.8% in rural EDs and 21.7% in urban EDs (p=0.326). Home healthcare was reported equally at both rural and urban EDs (69.6% and 60.9%, respectively). ED-based procedures (such as peripherally-inserted central catheter line placement or infusions), telemedicine, community paramedicine, and rapid specialist follow-up were all uncommon across both types of EDs.

### Barriers to Avoiding Admission

**Figure 1:**
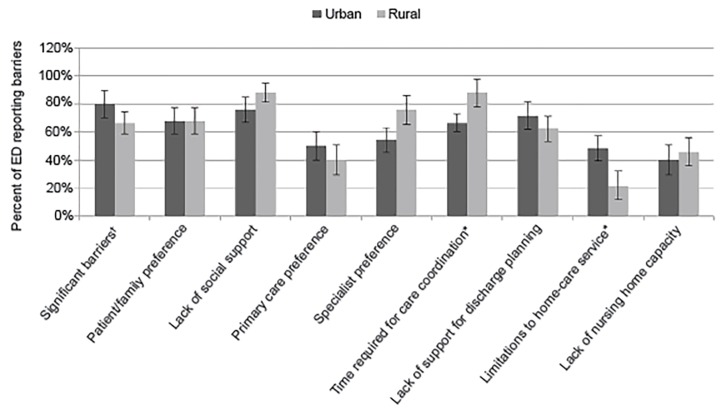
Barriers to avoidable admission reported by hospitals. Chi-squared analysis performed, with percent of rural and urban emergency departments (EDs) reporting barriers with associated 95% confidence intervals. Significant barriers defined as hospitals reporting the presence of five or more of the eight answer choices (the majority). Significance is at the p=.05 level. Data reported from responses to Q14 in Appendix. *Indicates a statistically significant result. ^†^Significant barriers is defined as the presence of five or more of the eight answer choices as this would represent a majority of barriers being selected.

Overall, barriers were high across all sites, with 74.2% of rural and 80.0% of urban sites reporting at least one barrier. Commonly reported barriers to avoiding admission in both rural and urban EDs included lack of social support (88.0% and 76.0%, p=0.27), patient/family preference (68.0% and 68.0%, p=1.0), primary care preference (40.0% and 50.0%, p=0.48), and specialist preference (76.0% and 54.2%, p=0.13) (Figure 1). Rural EDs faced more barriers than urban EDs for time required for care coordination (88.0% vs 66.7%, p=0.05) and fewer barriers to home care (21.7% vs 48.0%, p=0.05).

### Influence on ED Provider’s Decision to Admit

Rural EDs reported low levels of primary care (36% vs 56%, p=0.16) and specialist influence on their decision to admit (28% vs 56%, p=0.04) when compared to urban EDs. Overall, few sites reported that social workers, care managers, physical therapy and ED-based pharmacy had influence on the decision to admit, regardless of location.

### Does Urban-Rural Status Predict Ability to Reduce Low-Value Care?

**Figure 2:**
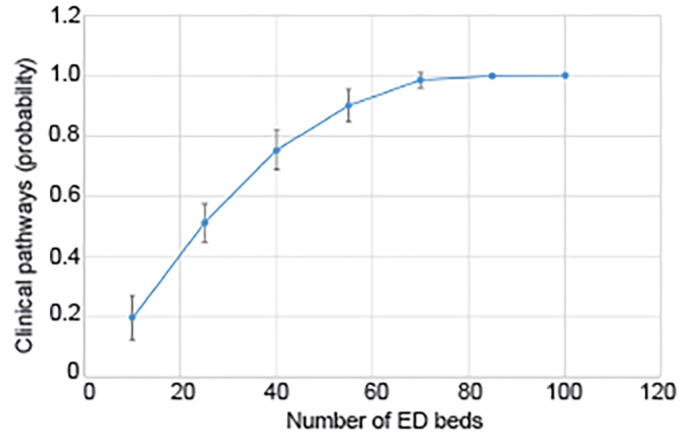
Adjusted proportion of emergency departments (ED) reporting clinical pathways. Error bars show 95% confidence intervals. Adjusted for presence of timely outpatient primary care follow-up, timely outpatient specialty care follow-up, outpatient resources (examples, wound care, or home healthcare), and presence of significant barriers to avoiding admission: defined as hospitals reporting the presence of five or more of the eight answer choices (the majority).

In our unadjusted multivariable analysis, rurality did not predict presence of clinical pathways; 51.5% (95% confidence interval [CI], 21.5–55.8) of urban and 38.7% of rural (95% CI, 34.4–68.5) had clinical pathways (p=0.3). After adjustment, the relationship remained non-significant (Table 3), although ED volume (number of beds) and average ED boarding time were significant. Of note, given the expected relationship between rurality and ED size, we did evaluate for multicollinearity; the variance inflation factor of 2.03 and indications of multicollinearity were not found. Adjusted predicted proportions showed a non-significant difference between the proportion of urban (40.9%, 95% CI, 23.9–57.9) and rural (59.4%, 95% CI, 46.1–72.8) EDs having clinical pathways after accounting for covariates. We further explored the relationship between ED size, as measured as the number of ED beds, and presence of clinical pathways while adjusting for urban/rural status. We found that each additional ED bed increased the likelihood of having a clinical pathway by 12.3%; for an ED with 25 beds the predicted probability of having clinical pathways was 51.1% and greater than 98% for those with 70 beds or greater (Figure 2). Thus, the relationship between the ED volume was predictive of the presence of a clinical pathway rather than rurality.

## DISCUSSION

As EDs are the primary source of acute hospitalizations in the United States (U.S.), they are positioned to link patients to alternative outpatient management strategies. However, the decision to hospitalize a patient is complex, and requires efficient, safe, and cost-effective outpatient care options for these alternative opportunities to be considered by ED providers and to be effective for patients. This survey of Michigan ED leaders regarding their local practices and resources demonstrates that about half of responding hospitals have clinical pathways to guide admission and discharge decisions. Yet despite the presence of standardized programs such as home healthcare or observation units to reduce avoidable admissions, most also reported significant barriers to discharging patients home from the ED, such as lack of social support, patient/family preference, and primary care and specialist preferences. As a result, regardless of location, both rural and urban EDs confront challenges to reducing avoidable hospitalizations even when clinical pathways exist.

In our analysis on the influence of rurality on our outcomes, we found that location did not predict the presence of clinical pathways, follow-up with primary or specialist care, barriers to avoiding admission, or presence of/access to outpatient resources. Instead, we found that as ED volume increased, so did the probability of having clinical pathways – indicating that larger EDs are more likely to use such pathways, regardless of location. This finding is consistent with literature demonstrating that clinical decision tools and pathways are more likely to be found in higher-volume EDs.^19^ In addition, while we found that geographic differences in the presence of services, programs, and barriers exist, rural EDs demonstrated robust efforts and appear to have services available to facilitate reducing avoidable hospitalizations.

### Connecting to Outpatient Care

Regardless of location, the perceived availability of primary and specialist care follow-up was low, indicating ongoing challenges related to fragmentation of care, particularly with respect to unscheduled acute care within the U.S. health system. This finding is consistent with trends demonstrating that fewer than half of acute care visits are managed by a patient’s personal physician; a growing share is now taking place in the ED^4^ with EDs increasingly supporting primary care practices to provide rapid, complex diagnostic work-ups, as well as after-hours demand for care.^20^ While this evolution in location of care is well documented, little research has been done to explore current patterns and barriers to emergency and primary care physician communication and coordination.^21^

As primary care continues to build capacity, partnering with local EDs in their efforts and in decision-making around admission or discharge will be important to overall success.^6^ While rural primary care practices may face barriers to care delivery due to lack of a robust primary care and specialist staffing pool as well as limited economies of scale,^15^ our data show potential for greater primary care availability in the rural setting. This may indicate that ED-primary care communication is easier within smaller communities with closer personal connections, and rural ED-primary care collaborations may be one model by which to improve rural population health.^22^

### Presence of Programs to Reduce Avoidable Hospitalizations

With the majority of sites reporting the presence of programs designed to avoid low-value hospitalizations, it appears that Michigan EDs are embracing efforts to reduce avoidable admissions. Some geographic variation exists, with greater awareness of wound care services and extended care facilities available to respondents in the rural setting, whereas observation units are more likely to be available to EDs found in the urban setting. This may reflect the needs of rural populations, which are traditionally older with multiple chronic conditions – both of which would require access to skilled nursing, wound care, and rehabilitation facilities. Home healthcare was consistently highly available to respondents from all EDs, matching national trends toward expanding home health services to support outpatient management strategies and meet the needs of an aging U.S. population.^23^ The second most reported service was observation units, with over 50% of urban EDs and 35% of rural EDs indicating presence of an ED observation unit. As urban hospitals are usually higher volume than rural, our finding is consistent with literature demonstrating that observation units are more commonly found in higher volume hospitals.^24^ While lower rates of observation units in rural EDs may reflect less perceived need or interest, the finding that one-third of rural EDs report their presence speaks to the penetration of this model of care in avoiding admission. While cost savings and perceived effectiveness of observation units by ED providers have been demonstrated,^25^,^26^ the impact of clinical pathways to improve patient outcomes while decreasing hospitalizations has not been rigorously studied and has been limited by variable implementation strategies and suboptimal research designs.^27^

It is unknown if ED providers routinely rely on home health, wound care, and care facilities as an alternative to hospitalization. The utilization of these resources was not studied in our survey, and future work should determine if presence and use are related. Further, these services are time consuming to arrange (over 70% of respondents indicated time required for care coordination and lack of support for discharge planning as a barrier) and may only be available to ED providers during business hours, limiting their impact. Finally, admission may be the only safe course of action for patients with complex social history or limited social support.

### Barriers

Barriers to avoiding low-value admissions were reported across all EDs, highlighting social and community challenges that extend beyond the ED setting. The least reported barrier was for rural EDs and home healthcare (21.7%), and the remaining barriers were present according to greater than 40% of respondents regardless of location. Remarkably, greater than 75% of all EDs reported lack of social support as a barrier to reducing avoidable admissions, followed by over 65% reporting family preference as a barrier. At present, the role for EDs in addressing issues of social isolation and home environment is limited. While there is a movement in the U.S. healthcare system to encourage primary care to address social determinants of health^28^ - or conditions in which people are born, grow, live, work and age – the success of this approach is unknown. EDs can play a role in identifying patients with significant social needs; however, this would require additional support since one of the other greatest barriers identified in our survey was the time required for care coordination and lack of support for discharge planning in the ED setting of care. Future work exploring patient and family needs would be helpful in understanding why a hospital admission is preferred and what services and support are critical in addressing these barriers.

## LIMITATIONS

There are several limitations to our study. First, it may suffer from response bias, as approximately half of Michigan EDs did not complete the survey. This could have been from a lack of interest in the topic, inadequate time to complete the survey, or improper selection of a contact person who felt comfortable answering these questions. While the results cannot be generalized to other states and environments, we did obtain a diverse set of responses from an important range of ED practice settings. Further, with equal representation between urban and rural sites, the validity of the comparison is strengthened despite the overall response rate. Respondents may also have been from “higher functioning” EDs or those with highly motivated administrators who have put robust efforts toward avoiding hospitalization or EDs that perceive avoiding hospitalization as important, even if not successful. The survey results suffered from missing data, as not all sites answered all the questions; however, the missing data appeared equally distributed between urban and rural EDs. Finally, our overall small sample size likely prevented us from detecting statistically and clinically important differences between sites, as several p-values approached significance.

## CONCLUSION

Both rural and urban EDs have an important role to play in reducing low-value hospitalizations but confront significant barriers to accomplishing this goal. In particular, a key obstacle universally identified was in connecting patients to timely, outpatient follow-up care, which could be bolstered by better integrating local EDs into patient-centered medical home efforts. While both urban and rural EDs in our study have implemented clinical pathways, the high prevalence of barriers and lack of connections to primary and specialty physicians limit the potential for their success without additional resources to build and strengthen alternatives to admission programs.

## Supplementary Information



## Figures and Tables

**Table 1:** Characteristics of participating emergency departments with associated descriptive statistics.

Characteristics	All EDs (n=64)	Urban EDs (n=33)	Rural EDs (n=31)
ED bed number (median [IQR])	20 [9–34]	34 [26–50]	9.5 [6–14]
Annual ED visit number (median [IQR])	26,413 [11,852–57,500]	57,000 [42,000–72,000]	12,061 [6,850–20,128]
Emergency physicians are hospital employees (average %)*	22.2%	17.4%	27.3%
Estimated ED boarding time (average min, [SD])	96.8 [74.7]	123.6 [84.1]	70.1 [53.7]

*IQR*, interquartile range; *ED*, emergency department.

* Number of hospitals reporting their emergency physicians are hospital employees (not a contracted physician group).

**Table 2:** Hospitals reporting presence of pathways and programs to prevent or reduce avoidable admissions.

Clinical pathways	Urban EDs, proportion (95% CI)	Rural EDs, proportion (95% CI)	P value
Overall presence	66.7% (48.6, 80.9)	74.2% (55.6, 86.9)	0.51
Home health	60.9% (39.2, 78.9)	69.6% (47.3, 85.3)	0.54
Wound care*	33.3% (19.1, 51.5)	62.5% (41.3, 79.8)	0.03
Extended care facility*	21.4% (9.6, 41.1)	52.2% (31.6, 71.9)	0.02
Primary care follow-up	21.7% (8.9, 44.0)	34.8% (17.8, 56.8)	0.33
Observation units*	51.5% (34.4, 68.3)	35.4% (20.4, 54.1)	0.04

*ED*, emergency department; *CI*, confidence interval.

Chi-squared analysis performed with percent of rural and urban EDs who report such pathways displayed.

* Indicates statistically significant results; significance is at the p=.05 level.

**Table 3:** Selected characteristics of emergency departments (ED) evaluated as predictors of the presence of clinical pathways; adjusted odds ratios with associated confidence intervals and p-values are reported.

Predictors	AOR (95% CI)	P value
Rurality	0.13 (0.01, 5.67)	0.29
Outpatient resources	0.63 (0.06, 6.97)	0.71
Significant barriers	0.30 (0.06, 1.52)	0.15
PCP follow-up	1.68 (0.14, 20.7)	0.69
Specialist follow-up	1.19 (0.15, 9.46)	0.87
Employment-type	0.02 (0.00, 1.69)	0.08
Boarding time	0.96 (0.93, 0.99)	0.01
ED bed number	1.2 (1.02, 1.42)	0.03

*AOR*, adjusted odds ratio; *CI*, confidence interval; *PCP*, primary care physician.
