# Supplementary material for: Are Rural and Urban Emergency Departments Equally Prepared to Reduce Avoidable Hospitalizations?
Source: West J Emerg Med. 2019 Apr 16;20(3):477–84. doi: 10.5811/westjem.2019.2.42057 (PMC6526889; doi:10.5811/westjem.2019.2.42057)
Supplement: Supplementary file 1 [file wjem-20-477-s001.docx]

| **Appendix.** MEDIC Environmental Scan Survey.  **Q1 Understanding Alternatives to Hospitalization after an ED Visit** | | | |  |  |  |  |  |  |  |  |  |  |
| --- | --- | --- | --- | --- | --- | --- | --- | --- | --- | --- | --- | --- | --- |
| We are conducting a study that seeks to better understand hospital emergency departments and efforts to connect patients with outpatient services aimed at reducing avoidable hospital admissions. | | | | |  |  |  |  |  |  |  |  |  |
| We are reaching out to leadership at all emergency departments in the state of Michigan to gather their responses to this brief survey.  We estimate this survey will take less than 15 minutes to complete. | | | | | | | | | | | | | |
|  |  |  |  |  |  |  |  |  |  |  |  |  |  |
| **Q2 Is your emergency department based in a hospital or a free standing facility?** | | | | | |  |  |  |  |  |  |  |  |
| o Hospital Based Emergency Department (2) | | |  |  |  |  |  |  |  |  |  |  |  |
| o Free Standing Emergency Department (1) | |  |  |  |  |  |  |  |  |  |  |  |  |
| o Urgent Care Center (4) |  |  |  |  |  |  |  |  |  |  |  |  |  |
|  |  |  |  |  |  |  |  |  |  |  |  |  |  |
| **Q3 For each of the following populations, please indicate the number of patients that visited and were admitted at your ED in 2015.** | | | | | | | | | |  |  |  |  |
| Please consider any patient under observation, with a disposition to another hospital unit, or inpatient status to have been admitted. | | | | | | | | |  |  |  |  |  |
| Please enter "N/A" if your hospital ED does not admit the listed population or "U" if the rate is unknown. | | | | | |  |  |  |  |  |  |  |  |
| o Total adult (18+) ED visits (1) ________________________________________________ | | | | | |  |  |  |  |  |  |  |  |
| o Total adult (18+) ED ADMITS (3) ________________________________________________ | | | | | |  |  |  |  |  |  |  |  |
| o Total child (<18) ED visits (2) ________________________________________________ | | | | | |  |  |  |  |  |  |  |  |
| o Total child (<18) ED ADMITS (4) ________________________________________________ | | | | | |  |  |  |  |  |  |  |  |
|  |  |  |  |  |  |  |  |  |  |  |  |  |  |
| **Q4 For each of the following populations, please indicate the estimated percent of total patients that were admitted in 2015.** | | | | | | | | |  |  |  |  |  |
| Please consider any patient with a disposition to another hospital unit, under observation, or inpatient status to have been admitted. | | | | | | | | |  |  |  |  |  |
| *Please enter "N/A" if your hospital ED does not admit the listed population or "U" if the rate is unknown.* | | | | | | |  |  |  |  |  |  |  |
|  | Estimated percent patients admitted in 2015 (2) |  |  |  |  |  |  |  |  |  |  |  |  |
| Children (< 18 years) (1) |  |  |  |  |  |  |  |  |  |  |  |  |  |
| Adults (18+ years) (2) |  |  |  |  |  |  |  |  |  |  |  |  |  |
|  |  |  |  |  |  |  |  |  |  |  |  |  |  |
| **Q5 How do you think your hospital's administration views the ED-based admission rate for children and adults?** | | | | | | | |  |  |  |  |  |  |
|  | Appropriate (4) | Too Low (2) | Too High (1) |  |  |  |  |  |  |  |  |  |  |
| Children (< 18 years) (4) | o | o | o |  |  |  |  |  |  |  |  |  |  |
| Adults (18+ years) (5) | o | o | o |  |  |  |  |  |  |  |  |  |  |
|  |  |  |  |  |  |  |  |  |  |  |  |  |  |
| **Q6 In your experience, how long does a typical patient wait, in MINUTES, from hospital admit time to ED departure time**  **for each of the following types of beds (i.e., boarding time)?** | | | | | | | | | | | | | |
| Please enter"N/A" if your hospital does not have one of the listed types of beds or "U" if the time is unknown. | | | | | | |  |  |  |  |  |  |  |
|  | Minutes spent waiting for a bed upon admission (2) |  |  |  |  |  |  |  |  |  |  |  |  |
| Telemetry (1) |  |  |  |  |  |  |  |  |  |  |  |  |  |
| Adult General Care (2) |  |  |  |  |  |  |  |  |  |  |  |  |  |
| Pediatric General Care (3) |  |  |  |  |  |  |  |  |  |  |  |  |  |
|  |  |  |  |  |  |  |  |  |  |  |  |  |  |
| **Q7 Some hospitals have resources to help the ED provider with their decision to admit a patient.**  **We would like to get an overall sense of the groups that CAN influence the decision to admit a patient from your ED to the hospital.** | | | | | | | | | | | | | |
| PRIOR to an ED provider's decision to admit a patient to inpatient or place a patient in observation, what additional services or  groups are involved in the decision to admit a patient to the hospital from the ED? | | | | | | | | | | | | | |
| *Please select one for each applicable group or service.* | | |  |  |  |  |  |  |  |  |  |  |  |
|  | All the time (1) | Frequently (2) | Rarely (4) | None of the time (5) |  |  |  |  |  |  |  |  |  |
| Care Manager (13) | ▢ | ▢ | ▢ | ▢ |  |  |  |  |  |  |  |  |  |
| Utilization Review (20) | ▢ | ▢ | ▢ | ▢ |  |  |  |  |  |  |  |  |  |
| Social Worker (14) | ▢ | ▢ | ▢ | ▢ |  |  |  |  |  |  |  |  |  |
| Physical Therapist (15) | ▢ | ▢ | ▢ | ▢ |  |  |  |  |  |  |  |  |  |
| ED Based Pharmacist (16) | ▢ | ▢ | ▢ | ▢ |  |  |  |  |  |  |  |  |  |
| Primary Care - PCP (17) | ▢ | ▢ | ▢ | ▢ |  |  |  |  |  |  |  |  |  |
| Specialist (18) | ▢ | ▢ | ▢ | ▢ |  |  |  |  |  |  |  |  |  |
| Other (7) | ▢ | ▢ | ▢ | ▢ |  |  |  |  |  |  |  |  |  |
|  |  |  |  |  |  |  |  |  |  |  |  |  |  |
| **Q8 AFTER an ED provider has decided to admit a patient, what criteria does your institution use to determine the**  **level of care (i.e., inpatient or observation status)?** | | | | | | | | | | | | |  |
| o Interqual (8) |  |  |  |  |  |  |  |  |  |  |  |  |  |
| o Milliman (9) |  |  |  |  |  |  |  |  |  |  |  |  |  |
| o Hospital developed criteria (10) | |  |  |  |  |  |  |  |  |  |  |  |  |
| o Other (11) ________________________________________________ | | | | |  |  |  |  |  |  |  |  |  |
| o None (12) |  |  |  |  |  |  |  |  |  |  |  |  |  |
| o I don't know (13) |  |  |  |  |  |  |  |  |  |  |  |  |  |
|  |  |  |  |  |  |  |  |  |  |  |  |  |  |
| **Q10 Many EDs have implemented pathways and protocols to guide admission decisions for specific conditions**  **or specific patient populations.** | | | | | | | | | | |  |  |  |
| Does your ED have any such pathways or protocols? | | |  |  |  |  |  |  |  |  |  |  |  |
| o Yes (1) |  |  |  |  |  |  |  |  |  |  |  |  |  |
| o No (2) |  |  |  |  |  |  |  |  |  |  |  |  |  |
| o Unsure (3) |  |  |  |  |  |  |  |  |  |  |  |  |  |
|  |  |  |  |  |  |  |  |  |  |  |  |  |  |
| **Q11 What clinical pathways or protocols does your ED have to facilitate discharge or avoid inpatient admission?** | | | | | | | |  |  |  |  |  |  |
| Of the example items listed below, please select all of the conditions with clinical pathways or protocols relevant to your ED.  Please also share with us any that are not on the list provided. | | | | | | | | | | | | |  |
| ▢ Asthma (1) |  |  |  |  |  |  |  |  |  |  |  |  |  |
| ▢ Atrial Fibrillation (2) |  |  |  |  |  |  |  |  |  |  |  |  |  |
| ▢ Bronchiolitis (3) |  |  |  |  |  |  |  |  |  |  |  |  |  |
| ▢ Cellulitis (4) |  |  |  |  |  |  |  |  |  |  |  |  |  |
| ▢ Chest Pain (5) |  |  |  |  |  |  |  |  |  |  |  |  |  |
| ▢ COPD (6) |  |  |  |  |  |  |  |  |  |  |  |  |  |
| ▢ Dehydration (7) |  |  |  |  |  |  |  |  |  |  |  |  |  |
| ▢ Head Injury (9) |  |  |  |  |  |  |  |  |  |  |  |  |  |
| ▢ Headache (10) |  |  |  |  |  |  |  |  |  |  |  |  |  |
| ▢ Pyelonephritis (14) |  |  |  |  |  |  |  |  |  |  |  |  |  |
| ▢ None of these (16) |  |  |  |  |  |  |  |  |  |  |  |  |  |
| ▢ Other (15) ________________________________________________ | | | | |  |  |  |  |  |  |  |  |  |
|  |  |  |  |  |  |  |  |  |  |  |  |  |  |
| **Q12 For each of the clinical pathways or protocols in your ED, please select all of the goals that may apply.** | | | | | | | |  |  |  |  |  |  |
|  | Standardized Observation Care (5) | Home with expedited referral to specialist (1) | Home with rapid follow up with PCP (3) | Other Services (7) | I don't know (4) |  |  |  |  |  |  |  |  |
| Asthma (x1) | ▢ | ▢ | ▢ | ▢ | ▢ |  |  |  |  |  |  |  |  |
| Atrial Fibrillation (x2) | ▢ | ▢ | ▢ | ▢ | ▢ |  |  |  |  |  |  |  |  |
| Bronchiolitis (x3) | ▢ | ▢ | ▢ | ▢ | ▢ |  |  |  |  |  |  |  |  |
| Cellulitis (x4) | ▢ | ▢ | ▢ | ▢ | ▢ |  |  |  |  |  |  |  |  |
| Chest Pain (x5) | ▢ | ▢ | ▢ | ▢ | ▢ |  |  |  |  |  |  |  |  |
| COPD (x6) | ▢ | ▢ | ▢ | ▢ | ▢ |  |  |  |  |  |  |  |  |
| Dehydration (x7) | ▢ | ▢ | ▢ | ▢ | ▢ |  |  |  |  |  |  |  |  |
| Head Injury (x9) | ▢ | ▢ | ▢ | ▢ | ▢ |  |  |  |  |  |  |  |  |
| Headache (x10) | ▢ | ▢ | ▢ | ▢ | ▢ |  |  |  |  |  |  |  |  |
| Pyelonephritis (x14) | ▢ | ▢ | ▢ | ▢ | ▢ |  |  |  |  |  |  |  |  |
| None of these (x16) | ▢ | ▢ | ▢ | ▢ | ▢ |  |  |  |  |  |  |  |  |
| Other (x15) | ▢ | ▢ | ▢ | ▢ | ▢ |  |  |  |  |  |  |  |  |
|  |  |  |  |  |  |  |  |  |  |  |  |  |  |
| **Q13 In your ED, hospital, or community, are there any standardized programs or services designed to**  **reduce avoidable inpatient hospital admissions?** | | | | | | | | | | | |  |  |
| *Please select all that apply.* |  |  |  |  |  |  |  |  |  |  |  |  |  |
| ▢ ED-based Procedures (e.g., infusions, PICC line placement) (6) | | | |  |  |  |  |  |  |  |  |  |  |
| ▢ Wound care (11) |  |  |  |  |  |  |  |  |  |  |  |  |  |
| ▢ Observation Unit(s) (4) |  |  |  |  |  |  |  |  |  |  |  |  |  |
| ▢ Extended Care Facility (5) |  |  |  |  |  |  |  |  |  |  |  |  |  |
| ▢ Home Health Care (2) |  |  |  |  |  |  |  |  |  |  |  |  |  |
| ▢ Same Day/Next Day access to Primary Care (7) | | |  |  |  |  |  |  |  |  |  |  |  |
| ▢ Community Paramedicine (19) | |  |  |  |  |  |  |  |  |  |  |  |  |
| ▢ Telemedicine (20) |  |  |  |  |  |  |  |  |  |  |  |  |  |
| ▢ ED Follow-up clinic (10) |  |  |  |  |  |  |  |  |  |  |  |  |  |
| ▢ Rapid Subspecialist Consultant follow-up Program (8) | | |  |  |  |  |  |  |  |  |  |  |  |
| Others (9) ________________________________________________ | | | |  |  |  |  |  |  |  |  |  |  |
|  |  |  |  |  |  |  |  |  |  |  |  |  |  |
| **Q14 In your community, what are the barriers to connecting ED patients with outpatient services that**  **could obviate the need for an inpatient hospital admission?** | | | | | | | | | | | |  |  |
|  |  |  |  |  |  |  |  |  |  |  |  |  |  |
|  | Barrier (1) | Not a Barrier (3) |  |  |  |  |  |  |  |  |  |  |  |
| Patient/family preferences for admission (4) | o | o |  |  |  |  |  |  |  |  |  |  |  |
| Lack of family and/or social support (17) | o | o |  |  |  |  |  |  |  |  |  |  |  |
| Primary care provider preferences for admission (5) | o | o |  |  |  |  |  |  |  |  |  |  |  |
| Specialty provider preferences for admission (6) | o | o |  |  |  |  |  |  |  |  |  |  |  |
| Time required for service coordination (38) | o | o |  |  |  |  |  |  |  |  |  |  |  |
| Lack of support for ED discharge planning (39) | o | o |  |  |  |  |  |  |  |  |  |  |  |
| Lack of timely outpatient primary care follow up (26) | o | o |  |  |  |  |  |  |  |  |  |  |  |
| Lack of timely outpatient specialty care follow up (9) | o | o |  |  |  |  |  |  |  |  |  |  |  |
| Limitations to home-care service availability (10) | o | o |  |  |  |  |  |  |  |  |  |  |  |
| Lack of nursing home capacity (11) | o | o |  |  |  |  |  |  |  |  |  |  |  |
| Other (13) | o | o |  |  |  |  |  |  |  |  |  |  |  |
|  |  |  |  |  |  |  |  |  |  |  |  |  |  |
| **Q15 How are the majority of physicians working in your hospital's ED employed?** | | | | | |  |  |  |  |  |  |  |  |
| *Please select one.* |  |  |  |  |  |  |  |  |  |  |  |  |  |
| o Hospital Employees (1) |  |  |  |  |  |  |  |  |  |  |  |  |  |
| o Contracted physician group employees (2) | | |  |  |  |  |  |  |  |  |  |  |  |
| o Don't know (3) |  |  |  |  |  |  |  |  |  |  |  |  |  |
| o Prefer not to answer (5) |  |  |  |  |  |  |  |  |  |  |  |  |  |
|  |  |  |  |  |  |  |  |  |  |  |  |  |  |
| **Q16 We are interested in learning more about your ED and your experiences regarding patient admissions.** | | | | | | | |  |  |  |  |  |  |
| We will be reaching out to a sample of participants to learn more about your ED's efforts to reduce avoidable hospital admissions. | | | | | | | |  |  |  |  |  |  |
| This survey and the subsequent phone interviews will help advance our knowledge regarding connecting ED patients to outpatient services. | | | | | | | | |  |  |  |  |  |
| If you are willing, please provide your contact information in the form below. | | | |  |  |  |  |  |  |  |  |  |  |
| o Name (1) ________________________________________________ | | | | |  |  |  |  |  |  |  |  |  |
| o Job Title (5) ________________________________________________ | | | | |  |  |  |  |  |  |  |  |  |
| o Hospital Unit (9) ________________________________________________ | | | | |  |  |  |  |  |  |  |  |  |
| o Hospital (2) ________________________________________________ | | | | |  |  |  |  |  |  |  |  |  |
| o Email Address (3) ________________________________________________ | | | | |  |  |  |  |  |  |  |  |  |
| o Work Phone (4) ________________________________________________ | | | | |  |  |  |  |  |  |  |  |  |
